# Salinity-influenced changes in the community and functional composition of zooplankton-associated bacteria in the lakes of Inner Mongolia

**DOI:** 10.3389/fmicb.2025.1529512

**Published:** 2025-06-17

**Authors:** Yuan Li, Qianhong Wang, Dongyi Chen, Xia Liu, Feizhou Chen

**Affiliations:** ^1^Key Laboratory of Lake and Watershed Science for Water Security, Nanjing Institute of Geography and Limnology, Chinese Academy of Sciences, Nanjing, China; ^2^College of Resources and Environment, University of Chinese Academy of Sciences, Beijing, China

**Keywords:** zooplankton-associated bacteria, *Moina mongolica*, salinity, 16S rRNA gene, community structure, ecological functions

## Abstract

In aquatic ecosystems, bacteria often reside on the surface or in the gut of zooplankton to play an indispensable role. Salinity is a key factor influencing the structure and functional composition of aquatic bacterial communities; however, its impact on zooplankton-associated bacteria (ZA) remains unclear. To address this knowledge gap, we conducted a study using 16S rRNA gene amplicon sequencing to investigate the ZA of the cladoceran *Moina mongolica* from lakes in the Inner Mongolian Plateau with different salinity groups (Low salinity: 2‰–3‰, High salinity: 17‰). By annotating the sequencing data, we identified the community structure of ZA, and we used the FAPROTAX database to infer their functional potential. Statistical analyses revealed that salinity is a significant environmental factor shaping the community structure and inferred functional composition of ZA. Higher salinity reduced the diversity and abundance of ZA, which, in turn, affected the biochemical functions contributed by these bacteria. Our results suggest that under salinity stress, the community structure and inferred functional composition of zooplankton-associated bacteria are affected, which may influence the ecological role of zooplankton in saline lakes. This study provides new insights into the ecological functions of zooplankton in saline lakes under the context of climate change and human activity.

## 1 Introduction

Zooplankton and bacteria are vital components of aquatic ecosystems. Bacteria in water are often threatened by predation, harmful chemicals, and other hazards, so some bacteria typically live in the intestines or exoskeletons of zooplankton, as these microhabitats can offer them refuge from external threats (Hansen and Bech, 1996; Carman and Dobbs, 1997; Samad et al., 2020). Compared to the external environment, the intestinal environment offers anaerobic conditions and a richer supply of nutrients (Nagasawa and Nemoto, 1988; Pruzzo et al., 1996; Tang et al., 2010). For instance, *Vibrio* can thrive within zooplankton, achieving densities that are 100–10,000 times greater than those of free-living bacteria (FL) (Heidelberg et al., 2002). The bacterial communities in the intestinal tracts of zooplankton consist of both stable and transient communities. Transient communities are actively ingested by the host during feeding and subsequently excreted, while stable communities persist within the intestinal tract (Harris, 1993). Therefore, following intestinal emptying, the diversity of zooplankton-associated bacteria (ZA) may decrease, but a substantial number of bacteria typically remain (Tang et al., 2006; Grossart et al., 2009). This allows bacteria to continuously exchange between the water column and zooplankton; The microenvironments provided by zooplankton, such as the intestinal tract and the exoskeleton, differ in their physical and chemical properties, which can favor the enrichment of distinct bacterial groups (Delille and Razouls, 1994; Hansen and Bech, 1996; Samad et al., 2020). For example, the chitinous exoskeletons of zooplankton can act as a carbon source, attracting specific chemotactic bacteria to colonize their surfaces (Pruzzo et al., 1996; Erken et al., 2015).

Environmental factors such as food availability, temperature, antibiotic presence, and nutrient levels can lead to shifts in the community structure of ZA (Bickel and Tang, 2014; Akbar et al., 2020, 2021). These changes are primarily reflected in the diversity, abundance, and composition of dominant bacterial species. ZA can influence the ecological roles of their hosts within aquatic ecosystems. For example, the ZA from certain zooplankton species inhabiting six freshwater wetlands in West Bengal, India, were found to significantly enhance seed germination rate, root and shoot length, and vigor index of various crops such as cauliflower, cowpea, and tomato (Islam et al., 2024). As the bacterial community evolves, these ecological functions may also be altered, as shown by Tandon et al. (2020), who found that changes in bacterial community composition can lead to shifts in metabolic pathways, such as increased butyrate metabolism and biofilm formation which were identified as key functional markers in high-salinity lakes. Consequently, fluctuations in external environmental factors can influence the ecological roles of zooplankton by affecting the community structure of their associated bacteria.

Salinity serves as a significant environmental determinant influencing bacterial community structure (Lozupone and Knight, 2007; Herlemann et al., 2011; Ji et al., 2019). Both diversity indexes and biochemical functions of free-living bacterial communities are affected by salinity, which has been shown to significantly impact their community structure and anaerobic ammonium oxidation processes in aquatic environments (Bouvier and Del Giorgio, 2002; Meng et al., 2018; Tang et al., 2021). Furthermore, salinity affects the expression of element cycle functions. For instance, bacterially mediated nitrogen metabolism is inhibited in high-salinity soil environments (Li et al., 2021). In mangrove sediments, salinity exhibits a negative correlation with bacterial denitrification activity and the abundance of *nirK* and *nosZ* denitrification genes (Wang et al., 2018). Additionally, in wetland habitats, the abundance and community structure of nitrate-reducing bacteria are closely related to salinity (Franklin et al., 2017). However, the effects of salinity on the community structure and functions of ZA have been rarely studied. For instance, Dziallas et al. (2013) investigated the bacterial communities associated with copepods and found that these communities were distinct from free-living bacteria, with potential influences from environmental factors such as salinity.

The Inner Mongolia Plateau, located in northern China, is home to numerous lakes and abundant natural resources. These lakes encompass a variety of types and sizes, contributing to a diversity of aquatic ecosystems in the region. In recent years, however, climate change and human activities have led to the shrinking and salinization of many lakes (Herbert et al., 2015; Tao et al., 2015; Zhou et al., 2019), consequently resulting in reduced biodiversity within these aquatic ecosystems. The salinity differences among various lakes in Inner Mongolia provide a gradient suitable for research. By investigating zooplankton and associated bacteria in lakes with differing salinities and comparing with other environmental factors, we can evaluate whether salinity serves as the primary driver of ZA change in these communities.

Here, we hypothesize that increased salinity would reduce the diversity and abundance of ZA, and that salinity would alter the community structure of ZA, thereby changing their inferred functional composition. We conducted a bacterial community survey in lakes on the Inner Mongolia Plateau, categorizing the lakes into high-and low-salinity groups based on salinity levels. By comparing the differences in the ZA community structure and inferred functional composition between the two salinity groups, we aim to explore the impact of salinity on ZA. Additionally, through surveys of FL and PA, we controlled for the influence of environmental bacteria and food sources on ZA. Our study provides insights into the role of zooplankton in ecosystems under salinity stress and the response patterns of associated bacteria. Furthermore, it fills the knowledge gap regarding the relationship between salinity and zooplankton-associated bacteria.

## 2 Materials and methods

### 2.1 Study area and sample collection

**Figure 1:**
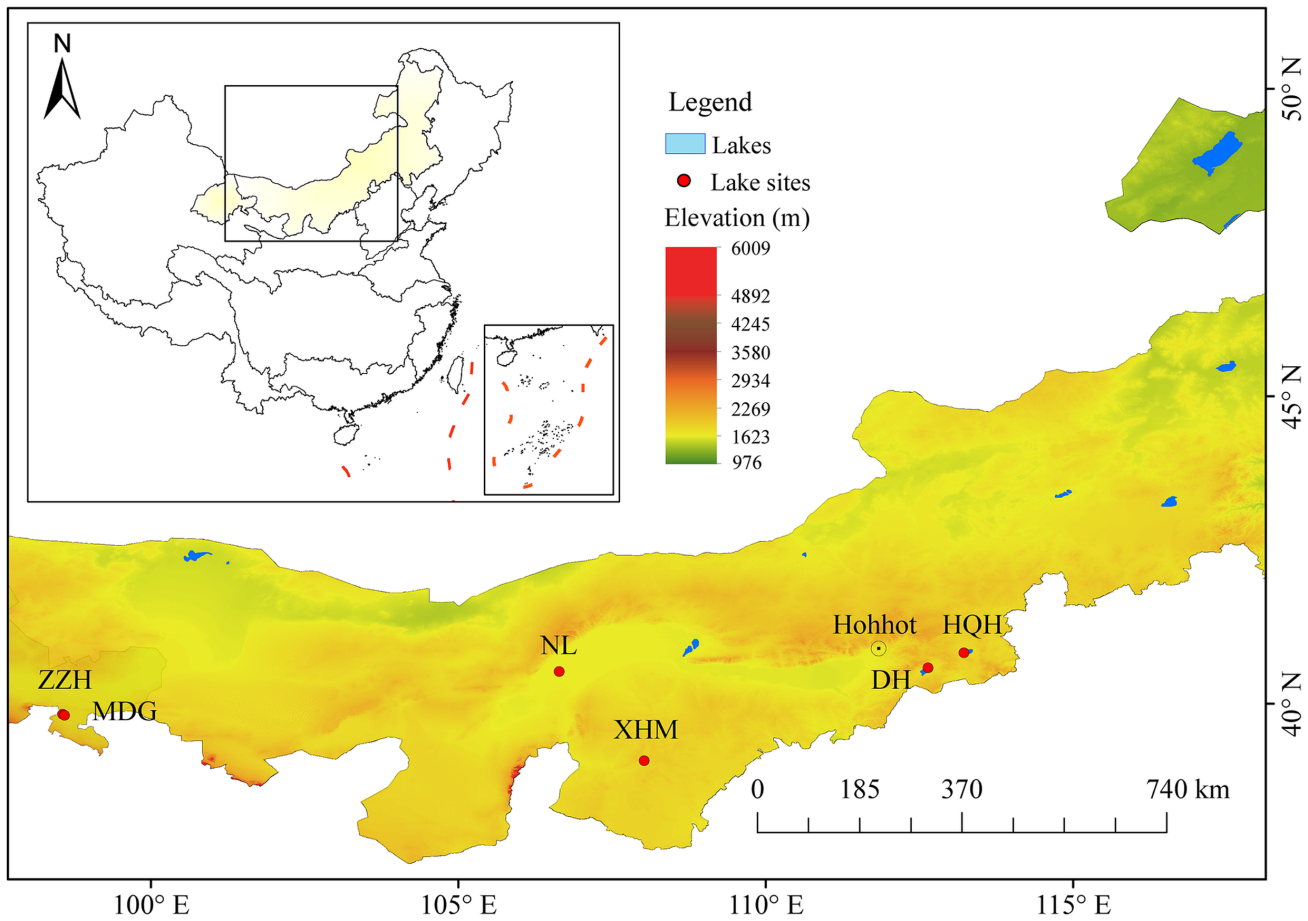
Satellite images of the lake sites located in the Inner Mongolian Plateau, China. The background elevation data were provided by Geospatial Data Cloud site, Computer Network Information Center, Chinese Academy of Sciences. (http://www.gscloud.cn). Administrative boundaries are based on public data provided by the National Geomatics Center of China (NGCC).

In 2019, we conducted an investigation across 18 lakes in the Inner Mongolia Lake District, where salinity ranged from 0.84‰ to 115‰. In lakes with salinity exceeding 17‰, only *Artemia* were found, with no other zooplankton present. In lakes with salinity below 17‰, we collected *Daphnia magna*, *Moina mongolica*, *Calanus* and *Cyclops*. Notably, *M. mongolica* was the only species found in six lakes across different salinity gradients. Among them, the Maodonggou Reservoir (MDG), Lake Nalin (NL), Lake Xiaohamarigetainor (XHM), and Lake Zhangzonghaizi (ZZH) were categorized as the low salinity group (salinity 2‰ - 3‰), while Lake Huangqihai (HQH) and Lake Daihai (DH) were classified as the high salinity group (salinity 17‰, Supplementary Table S1). ZZH and MDG are located in Jiuquan City, Gansu Province, while the remaining four lakes are situated in the Inner Mongolia Autonomous Region (Figure 1).

We established sampling sites in the pelagic area of each lake, with four sites in DH and NL, three in HQH, two in MDG and XHM, and one in ZZH. At each sampling point, geographic coordinates (longitude and latitude) and water depth were recorded using a handheld GPS and depth sounder. Zooplankton were collected at each site using a 64 μm mesh size zooplankton net and temporarily stored in 500 mL plastic bottles. In addition, surface, middle and bottom water were collected at each sites using a 5 L water sampler and then mixed in an open-top bucket. 10 L of the mixed water was placed in a lidded bucket and brought back to the laboratory along with the zooplankton samples.

In the laboratory, lake water was filtered through a 0.2 μm pore-size polycarbonate filter (Millipore, Billerica, MA, United States) to obtain sterile lake water. Zooplankton samples were sorted under a dissecting microscope to isolate *M. mongolica,* which were then placed in sterile lake water in a beaker for 2 h to allow them to empty their intestines. After this, the *M. mongolica* individuals were transferred to 2 mL cryogenic vials, which were then quickly frozen in liquid nitrogen to preserve their biological integrity. The vials were subsequently stored in a -80°C freezer for further use in the DNA extraction of zooplankton-associated bacteria (ZA).

Subsamples of lake water (150–300 mL) were pre-filtered through a 64 μm zooplankton net to exclude larger zooplankton, and then filtered through 5 μm and 0.2 μm pore-size polycarbonate filters for molecular analysis. The 5 μm filters were used for capturing particle-attached bacteria (PA), while the 0.2 μm filters were used for free-living bacteria (FL). The filters were stored at −80°C in the laboratory for DNA extraction. The remaining water, which was also filtered through a 0.2 μm polycarbonate filter, was used for immediate chemical analysis.

### 2.2 Measuring environmental parameters

Water depth (WD) was recorded for each sampling point. Water clarity (SD) was assessed using a transparency disk. A multi-parameter water quality tester (YSI 6600, Yellow Springs, OH, USA) was employed to measure water temperature (WT), pH, conductivity (Cond), salinity, dissolved oxygen (DO), and oxidation–reduction potential (ORP) at a depth of approximately 1 meter underwater.

Upon returning to the laboratory, we measured an additional six parameters from the water samples filtered through a 0.2 μm membrane, following standard methods (Jin and Tu, 1990): total nitrogen (TN), ammonia nitrogen (NH_4_-N), nitrate nitrogen (NO_3_-N), total phosphorus (TP), orthophosphate (PO_4_-P), and chlorophyll a (Chl-*a*).

### 2.3 Bacterial DNA extraction and sequencing data analysis

DNA extraction from each sample was performed using the MagaBio Soil Genomic DNA Purification Kit (MagaBio Soil/Feces Genomic DNA Purification kit). Primers 338F (5’-ACTCCTACGGGA GGCAGCAG-3′) and 806R (5’-GGACTACHVGGGTWTCTAAT-3′) (Klindworth et al., 2013) were utilized to target the bacterial rRNA V3-V4 region of ZA. For FL and PA, the bacterial rRNA V4 region was targeted using primer 515F (5’-GTGCCAGCMGCCGCGGTAA-3′) in conjunction with primer 806R (Caporaso et al., 2011).

PCR was performed using TakaRa Premix Taq^®^ Version 2.0 (TaKaRa Biotechnology Co., Dalia). The PCR mixture comprised 50 ng of DNA template, 10 μM of each primer, and 25 μL of 2x Premix Taq. A negative control was included during the PCR to monitor potential contamination. The reaction protocol included pre-denaturation at 94°C for 5 min, followed by 30 cycles of denaturation at 94°C for 30 s, annealing at 52°C for 30 s, and extension at 72°C for 30 s. A final extension step was performed at 72°C for 10 min, followed by stabilization at 4°C for 4 min. Library construction was carried out according to the standard protocol of the NEBNext® UltraTM II DNA Library Prep Kit for Illumina^®^ (New England Biolabs, USA). The constructed amplicon library underwent PE250 sequencing using the Illumina Nova 6,000 platform. Sliding window quality trimming of paired-end Raw Reads data (-W 4 -M 20) was performed using fastp (version 0.14.1) (Chen et al., 2018), taking into account the primer information at both ends of the sequence. The cutadapt software (Marcel, 2011) was utilized to remove primers and obtain paired-end Clean Reads after quality control. The usearch-fastq mergepairs (V10) (Arraiano-Castilho et al., 2020) tool was employed to filter out unmatched tags and generate the original spliced sequence (Raw Tags). Finally, fastp was used to perform sliding window quality trimming on the Raw Tags data to obtain effective splicing fragments (Clean Tags).

The UPARSE1 method was employed for OTU clustering, followed by the use of usearch-sintax to compare the representative sequence of each OTU with the SILVA database (version 138), aiming to obtain species annotation information (threshold set at 0.8, Edgar, 2013; Quast et al., 2012). OTUs annotated as chloroplasts or mitochondria, as well as those that could not be annotated to the kingdom level, were removed. Subsequently, the number of valid Tags sequences (No. of seqs) and the comprehensive OTU taxonomy information table (OTU-table) for each sample were obtained for further analysis. Additionally, OTUs with the lowest abundance (<10 reads) were eliminated.

Ultimately, we obtained OTUs representing 16 ZA samples, with the high-salinity group containing 7 samples (4 from DH and 3 from HQH) and the low-salinity group comprising 9 samples (2 each from MDG and XHM, 4 from NL, and 1 from ZZH). Additionally, the OTUs included 9 FL and PA samples, with 3 in the high-salinity group (1 from DH and 2 from HQH) and 6 in the low-salinity group (1 each from MDG and ZZH, and 2 each from NL and XHM).

### 2.4 Data analysis

A Venn diagram illustrating the number of ZA OTUs among different groups was plotted using the R package “VennDiagram” (version 1.6.0) (Chen and Boutros, 2011). Prior to α-diversity analysis, sequencing depth was normalized according to the minimum read count among samples (46,819 reads for ZA and 41,274 reads for FL and PA in this study). The α-diversity indices, including Shannon, Chao1, and Richness, were calculated using the R package “vegan” (version 2.6–4) (Dixon, 2003). Differences in α-diversity indices between groups were tested using t-tests. In addition, rarefaction curves based on the Richness index were plotted to assess whether the sequencing depth was sufficient to cover the majority of bacterial taxa in each sample. The step size of the rarefaction was set to 6,000 sequences to ensure the smoothness and comparability of the curves. The shape and plateau of the curves were used to evaluate whether sequencing saturation had been reached, thereby validating the reliability of the sequencing data. The OTUs data of ZA samples were Hellinger-transformed using “vegan,” and Bray-Curtis distance were then calculated. Principal coordinates analysis (PCoA) (Zhang et al., 2022) and PERMANOVA (Eckert et al., 2021) were conducted based on the Bray-Curtis distance.

Relative abundances were calculated at the phylum, class, and genus levels based on the OTUs data, and differences between groups were tested using the Wilcoxon rank-sum test. OTUs data were compared with the FAPROTAX to annotate the inferred functional composition of bacteria (Louca et al., 2016). The OTUs categorized by each functional group were automatically matched with the FAPROTAX_1.2.4 database^1^ online. Since FAPROTAX is a database that links taxonomic identity to functional potentials based on cultured representatives, the inferred functional composition predicted in this study reflects the potential metabolic capabilities of bacterial communities rather than direct experimental measurements of functional activity. A heatmap was generated using the R package “pheatmap” (version 1.0.12) (Kolde, 2019) to visualize the functional profiles of zooplankton-associated bacteria. Additionally, Kruskal–Wallis tests were used to assess differences in inferred functional abundance across groups. The STAMP software (version 2.1.3) (Parks et al., 2014) was used to perform Welch’s t-tests and generate plots of functional profiles across groups. In the results, ZA showed the top 15 functional categories with the smallest *p*-values, while all significantly different functions were displayed for FL and PA. Positive values indicate higher relative abundance in the high-salinity group, whereas negative values indicate higher abundance in the low-salinity group.

The Pearson correlations between environmental variables were calculated using the R package “ggcor^2^” (version 0.9.8). Additionally, Mantel tests were performed using the “vegan” to examine the correlations between bacterial community composition, inferred functional composition, and environmental factors. Highly collinear environmental variables identified through correlation analysis were removed from subsequent analyses.

To explore environmental variables significantly related to bacterial community composition and inferred functional composition, Canonical Correspondence Analysis (CCA) was conducted using the “vegan” (Borcard et al., 2011). Given that multiple environmental factors may influence bacterial communities, CCA was applied to assess the relative contribution of salinity compared to other environmental variables. To mitigate the influence of differing measurement scales, environmental data were standardized. Forward selection, using a Monte Carlo test with 999 permutations (Blanchet et al., 2008), retained only those variables that significantly contributed to explaining additional proportions of total variance (*p* < 0.05). Subsequently, the Variance Inflation Factors (VIF) of the filtered variables were calculated, and those with VIF values exceeding 10 (indicating strong collinearity) were eliminated. To further evaluate the relative importance of salinity in shaping bacterial communities, the Variation Partitioning Approach (VPA) was utilized, allowing us to quantify the proportion of variance explained by salinity in relation to other environmental factors.

All statistical analyses and visualizations were performed using R version 4.2.3^3^ and RStudio version 2023.12.0. Data visualization was conducted using the R packages “VennDiagram,” “ggplot2 (Wickham, 2016),” “pheatmap,” and “ggcor,” as well as the STAMP software.

## 3 Result

### 3.1 Alpha diversity of Bacteria

**Figure 2:**
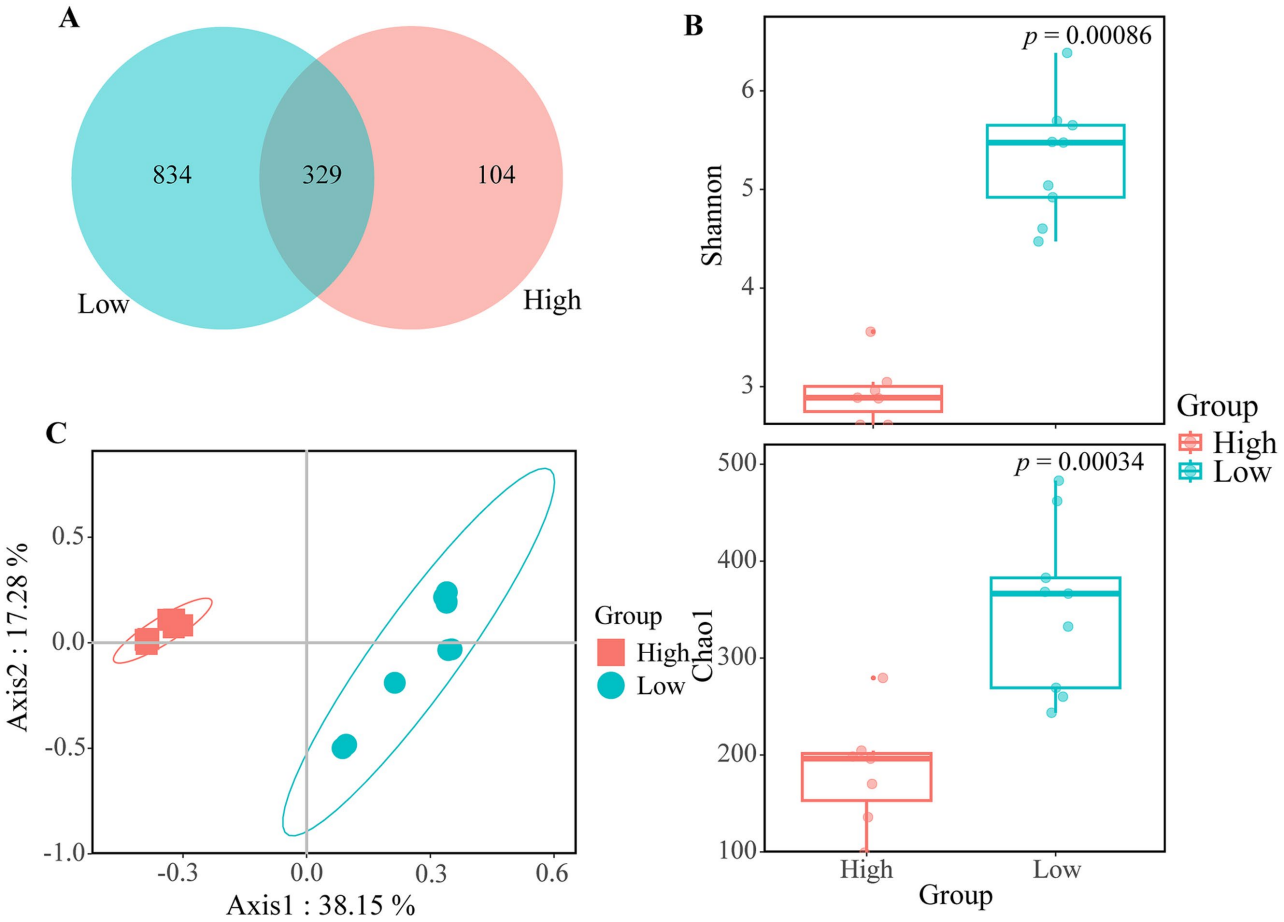
Differences in alpha- and beta-diversity between different groups in zooplankton-associated bacteria. **(A)** Venn diagram illustrating the distribution of bacterial OTUs. **(B)** Boxplot comparison of *α*-diversity as measured by the Shannon index and Chao1 index among high- and low-salinity lakes. *t*-test was conducted to assess the significance of differences. **(C)** Principal Coordinate Analysis (PCoA) comparing samples from high- and low-salinity group.

The 16 ZA samples yielded a total of 749,104 high-quality reads, averaging 46,819 reads per sample. These reads were classified into 1,267 OTUs, of which 8.6% were unique to the high-salinity group, 65.8% to the low-salinity group, and 27.3% were shared by both groups (Figure 2A). Similarly, the FL and PA samples produced 742,446 bacterial sequences, resulting in 2,405 OTUs.

To assess sequencing depth, rarefaction curve analysis was performed. For ZA samples, the curves approached an asymptote after 10,000 reads (Supplementary Figure S1A), indicating that most bacterial diversity was captured. In contrast, the FL and PA samples reached their asymptote after 40,000 reads (Supplementary Figure S1B). The sequencing coverage for the ZA samples was 99.9%, while the FL and PA samples reached 99.5%, indicating that nearly all bacterial sequences were sufficiently represented in the data.

In terms of α-diversity, the Shannon and Chao1 indices were significantly lower in the high-salinity group compared to the low-salinity group for ZA samples (Figure 2B). However, no significant differences in α-diversity were observed between high-and low-salinity lakes for FL and PA samples (Supplementary Figures S2A,B).

According to Bray–Curtis dissimilarity, the taxonomic distance between the two groups was clearly distinct. Specifically, the samples from the low-salinity group were more dispersed, while the samples from the high-salinity group were more tightly clustered (Figure 2C). PERMANOVA also revealed a significant difference in the community structure of ZA between the two groups (R^2^ = 0.47, *p* = 0.002).

### 3.2 Bacterial community composition

**Figure 3:**
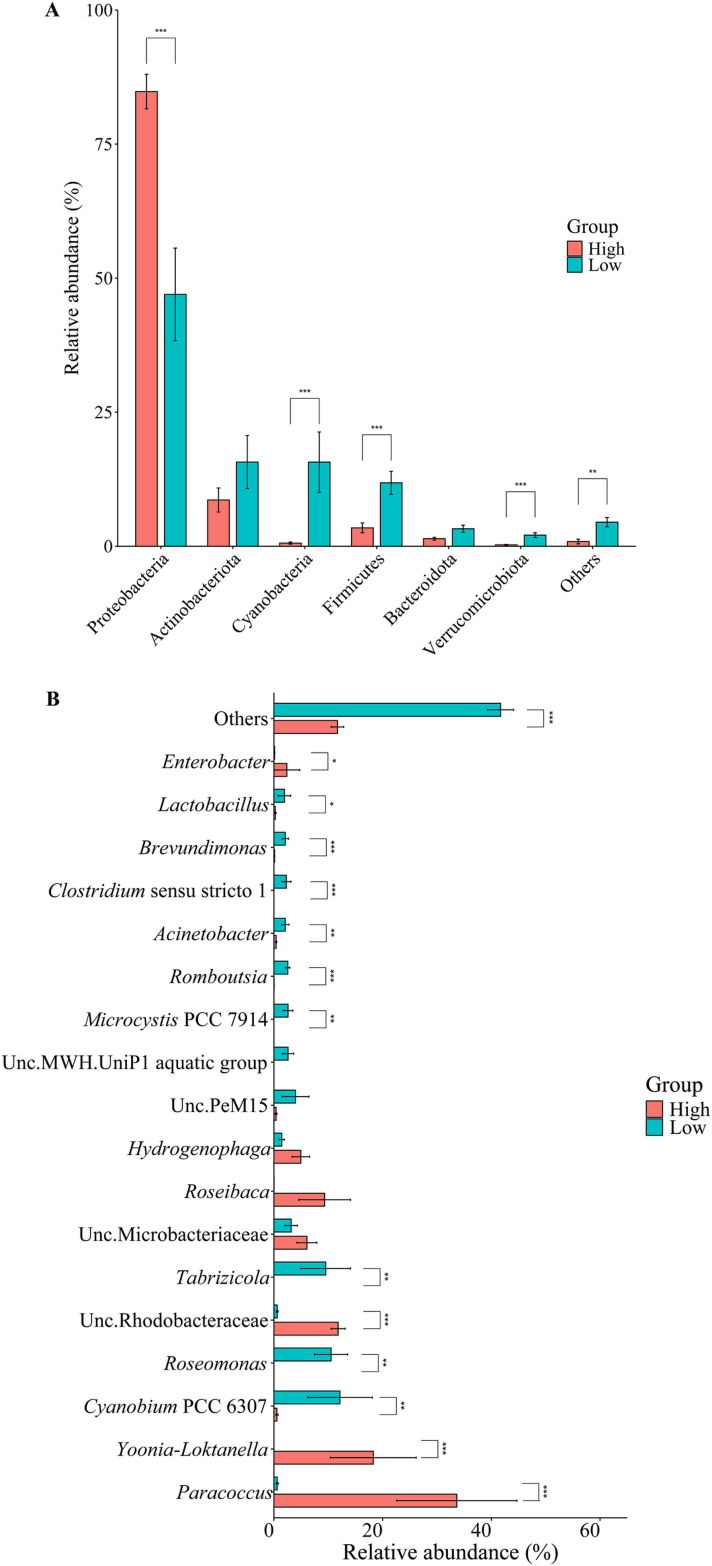
Community structure of zooplankton-associated bacteria. The bar plots display the relative abundance of community composition at the **(A)** phylum level (Top 6) and at the **(B)** genus level (Top 18). Analyses were conducted using Wilcoxon rank-sum tests. Significance levels: *p < 0.05, **p < 0.01, ***p < 0.001.

A total of 38 phyla were detected in the ZA samples, with 6 major phyla (relative abundance > 1%) identified: Proteobacteria, Actinobacteriota, Cyanobacteria, Firmicutes, Bacteroidota, and Verrucomicrobiota. Among these, the relative abundance of Proteobacteria in the high-salinity group (84.80% ± 3.21%) was significantly higher than that in the low-salinity group (46.97% ± 8.63%). In contrast, the relative abundance of Cyanobacteria, Firmicutes and Verrucomicrobiota in the low-salinity group were significantly higher than those in the high-salinity group (Figure 3A). There were 10 dominant classes in the ZA samples. Among them, the relative abundance of Alphaproteobacteria was significantly higher in the high-salinity group, whereas Cyanobacteria, Clostridia, and Verrucomicrobiae were much more abundant in the low-salinity group (Supplementary Figure S3). Among the 18 major genera (relative abundance > 1%) in the ZA samples, the relative abundances of 13 genera differed significantly between the groups (Figure 3B).

In the FL samples, a total of 27 phyla were detected, with 8 main phyla (relative abundance > 1%) identified: Proteobacteria, Actinobacteria, Cyanobacteria, Bacteroidetes, Verrucomicrobia, Firmicutes, Patescibacteria, and Planctomycetes. The relative abundance of Bacteroidetes in the high-salinity group was significantly higher than in the low-salinity group. Conversely, the relative abundances of Verrucomicrobia and Patescibacteria were significantly higher in the low-salinity group compared to the high-salinity group. The relative abundances of the other phyla did not show significant differences between the groups (Supplementary Figure S4A). There were 11 dominant classes in the FL samples. Among them, the relative abundance of Bacteroidia was significantly higher in the high-salinity group, whereas Verrucomicrobiae and Parcubacteria were significantly more abundant in the low-salinity group (Supplementary Figure S4B). Among the 19 major genera (relative abundance > 1%), 6 exhibited significant differences in relative abundance between the groups. Specifically, the relative abundances of DS001, *Acinetobacter*, *Sphingomonas* and *Chryseobacterium* were significantly higher in the high-salinity group compared to the low-salinity group, while LD29 had a significantly higher relative abundance in the low-salinity group compared to the high-salinity group. The relative abundances of the remaining genera did not differ significantly between the groups (Supplementary Figure S4C).

In the PA sample, a total of 33 phyla were detected, with 8 being predominant (relative abundance > 1%). Among these, the relative abundance of Cyanobacteria, Planctomycetes, and Patescibacteria were significantly higher in the low-salinity group compared to the high-salinity group. Conversely, the relative abundance of Bacteroidetes was significantly higher in the high-salinity group than in the low-salinity group (Supplementary Figure S5A). There were 13 dominant classes in the PA samples. Among them, the relative abundances of Bacteroidia and Nitriliruptoria were significantly higher in the high-salinity group, while Parcubacteria was significantly more abundant in the low-salinity group (Supplementary Figure S5B). Among the 17 major genera (relative abundance > 1%), *Cyanobium* PCC 6307 was the most dominant, with its relative abundance in the low-salinity group significantly higher than that in the high-salinity group. Following this, DS001 *and Paracoccus* had significantly higher relative abundances in the high-salinity group compared to the low-salinity group (Supplementary Figure S5C).

### 3.3 Bacterial inferred functional composition

**Figure 4:**
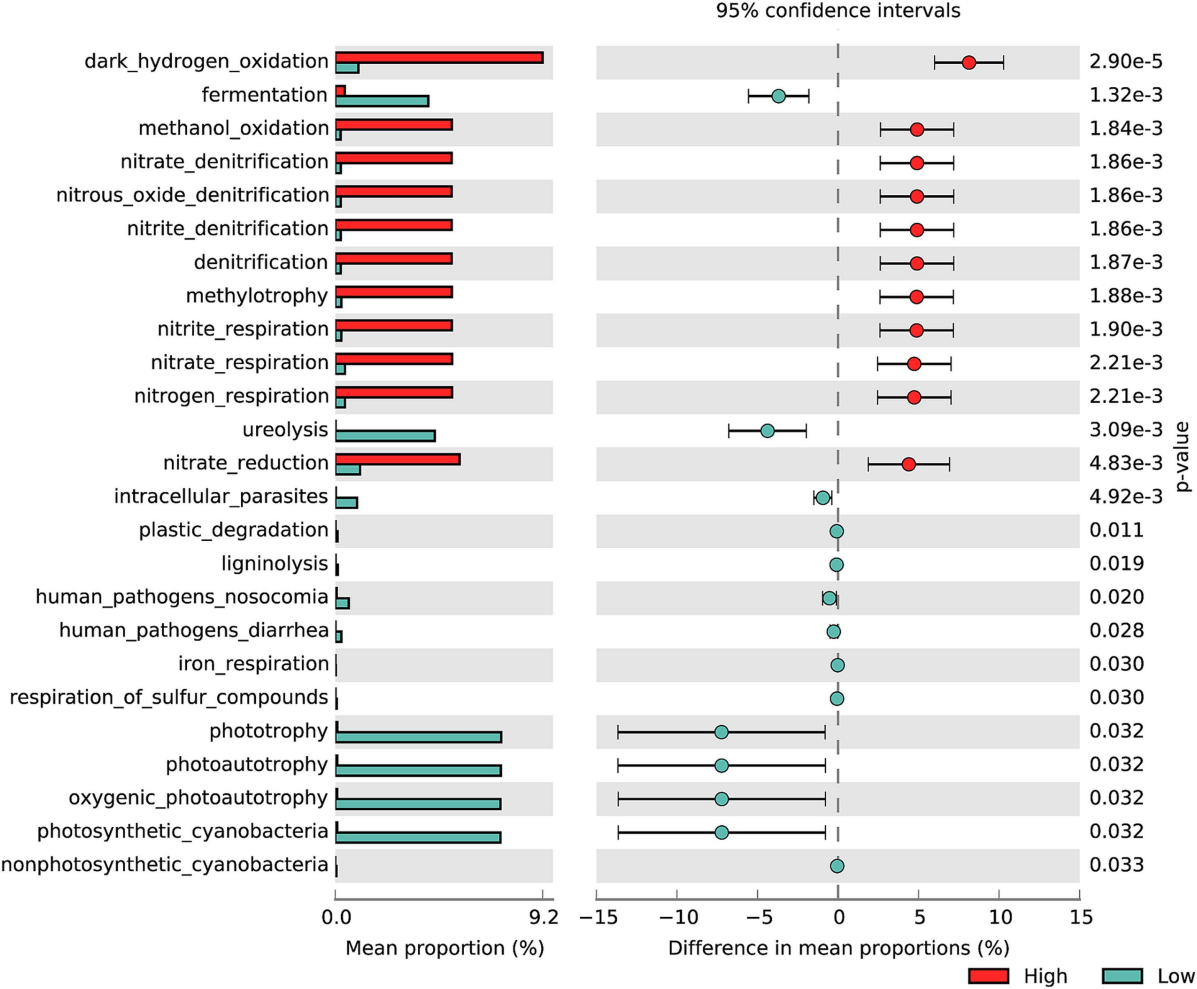
Difference in the functional distribution of zooplankton-associated bacteria based on FAPROTAX inferred function among high- and low-salinity groups. The analysis was conducted using Welch’s *t*-test.

The functional annotation of OTUs revealed a rich repertoire of metabolic functional types. In total, 626 out of 2,109 OTUs (30%) were assigned to at least one functional type in the ZA groups, representing 74 out of 92 functional types from the FAPROTAX 1.2.4 database. Among the identified functional types, 31 functions showed significant differences between the high-and low-salinity groups (Supplementary Figure S6). Specifically, denitrification-related functions (contributed by the genus *Paracoccus* and *Enterobacter*), dark hydrogen oxidation (contributed by the genus *Paracoccus*), and methanol oxidation (contributed by the genus *Paracoccus*) were significantly enriched in the high-salinity group. Conversely, fermentation (contributed by the genus *Romboutsia*), ureolysis (contributed by the genus *Roseomonas*), and photoautotrophy (contributed by the genus Microcystis PCC 7914 and *Cyanobium* PCC 6307) were significantly enriched in the low-salinity group (Figure 4).

In the FL groups, 347 out of 1,345 OTUs (26%) were assigned to at least one functional type, representing 49 out of 92 functional types. Functional enrichments with significant differences across various groups included phototrophy, oxygen-producing photoautotrophy, photosynthetic cyanobacteria, and photoautotrophy (contributed by the genus *Cyanobium* PCC 6307, Supplementary Figure S7A). In the PA groups, 449 out of 1757 OTUs (26%) were assigned to at least one functional type, representing 56 out of 92 functional types. The significantly enriched functions in the high-salinity group include nitrogen reduction, dark hydrogen oxidation, and methanol oxidation, all primarily contributed by the genus *Paracoccus*. In contrast, the low-salinity group showed significant enrichment in photoautotrophy, oxygen-producing photoautotrophy, and photosynthetic cyanobacteria, all primarily contributed by *Cyanobium* PCC 6307. (Supplementary Figure S7B).

### 3.4 Environmental drivers on community and inferred functional composition of ZA

**Figure 5:**
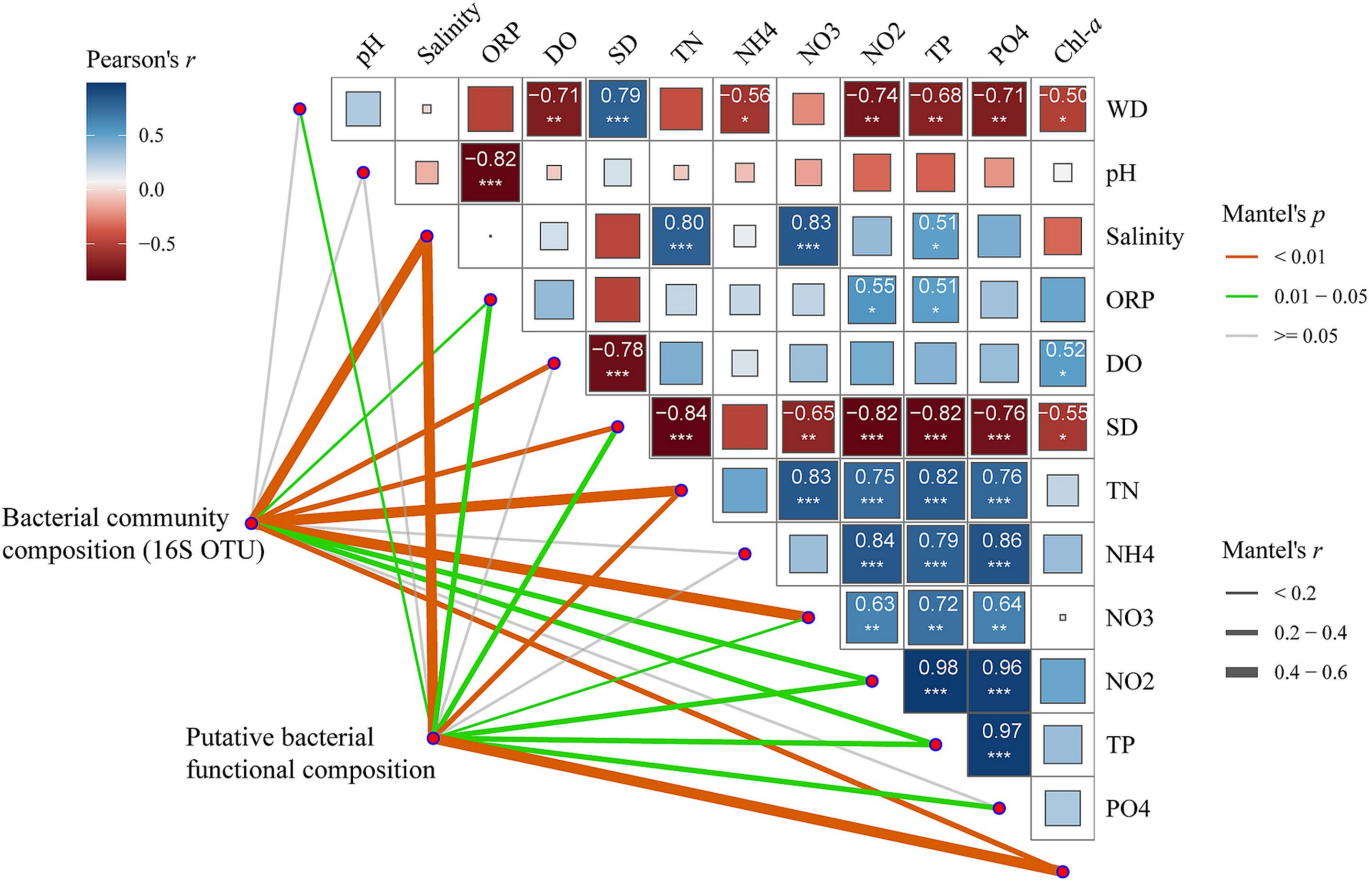
Pearson’s correlation coefficients among the main 12 environmental factors, bacterial community composition, and inferred functional composition of zooplankton-associated bacteria, assessed using Mantel tests. Block size and color indicate the value and direction of the correlation coefficients. Numbers represent *r* values. Asterisks denote significance levels: * *p*-value < 0.05, ** *p*-value < 0.01, *** *p*-value < 0.001. Line width corresponds to Mantel’s *r* statistic for the corresponding distance correlations, while line color denotes statistical significance.

The Mantel test indicated that community composition of ZA was correlated with salinity (*r* = 0.59, *p* < 0.001), TN (*r* = 0.45, *p* < 0.001) and NO_3_-N (*r* = 0.45, *p* < 0.001). Additionally, the inferred functional composition of ZA showed correlations with both salinity (*r* = 0.47, *p* < 0.001) and Chl-*a* (*r* = 0.43, *p* < 0.001, Figure 5).

**Figure 6:**
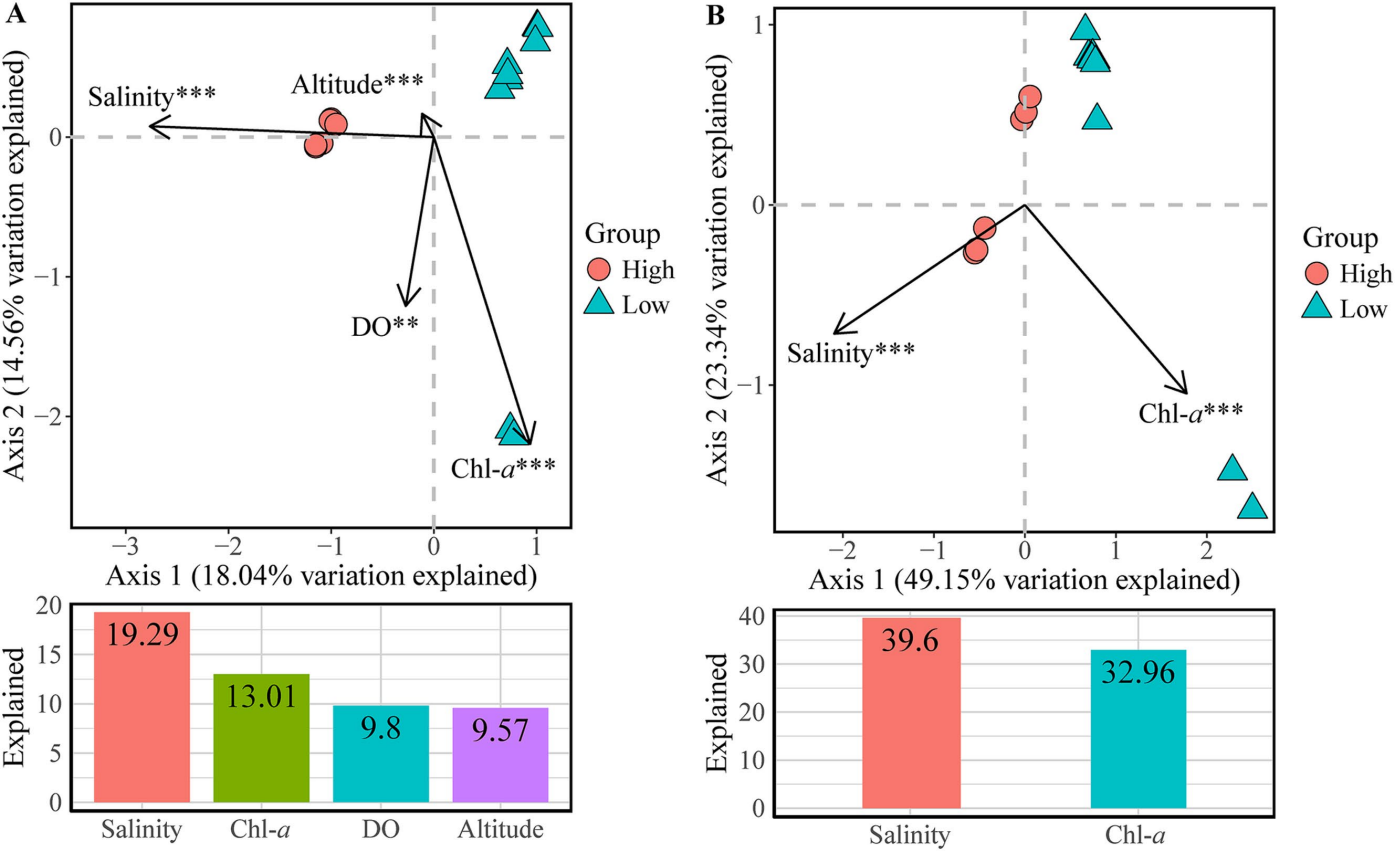
Canonical correspondence analysis (CCA) plots showing the significant environmental factors structuring variations in **(A)** bacterial community composition and **(B)** inferred functional composition of zooplankton-associated bacteria. Bar plots below each CCA panel show the variation explained by each factor. Significance levels: ** *p*-value < 0.01, *** *p*-value < 0.001.

The forward selection procedure in Canonical Correspondence Analysis (CCA) revealed that the variation in the community composition of ZA was significantly explained by four environmental factors: salinity, Chl-*a*, DO and altitude (Figure 6A). Together, these variables accounted for 51.7% of the variation, leaving 48.3% unexplained. Notably, salinity had the highest explanatory rate, contributing 19.29%.

According to the CCA forward selection process, Salinity and Chl-*a* significantly affected the inferred functional composition of ZA (Figure 6B). Together, these variables collectively explained 72.6% of the variation, leaving 27.4% unexplained. Specifically, salinity accounted for 39.6% of the variation, while Chl-*a* explained 32.96%.

## 4 Discussion

Our study is the first to reveal the effects of salinity on both the zooplankton-associated bacterial (ZA) community and inferred functional composition. The diversity and abundance of ZA in the high-salinity group (17‰) was significantly lower than in the low-salinity group (2‰-3‰). However, we observed no significant differences in the diversity and abundance of the free-living bacteria (FL) and particle-attached bacteria (PA) between the two groups. Previous studies have noted a decreasing trend in FL in lakes of Inner Mongolia (salinity range of 0.14‰ –11.36‰, Tang et al., 2021) and the Tibetan Plateau (salinity range of 0.14‰ –118.07‰, Ji et al., 2019), as well as in both FL and PA in the Xinglinwan Reservoir (salinity range of 1.1‰ –8‰, Yan et al., 2024). This discrepancy may be attributed to the absence of freshwater lakes in our study, as noted by Tang et al. (2021) and the differing salinity ranges reported by Ji et al. (2019) and Yan et al. (2024). This suggests that the difference in FL and PA diversity and abundance become more pronounced as salinity increases. In our studied lakes, no cladocerans or copepods were found when salinity exceeded 17‰. Unlike FL and PA, ZA diversity exhibited significant differences even within the smaller salinity ranges, potentially reflecting the complex physiological processes of zooplankton and their impact on ZA.

The CCA and VPA results indicated that among all environmental factors significantly influencing bacterial community composition (BCC) and inferred functional composition (BFC), salinity explained the greatest proportion of the variance. Therefore, salinity could be identified as the primary driving factor shaping both the ZA community structure and its functional potential. Among the dominant genera in the ZA community, three genera, *Paracoccus*, *Yoonia*-*Loktanella*, and *Enterobacter*, exhibited significantly higher relative abundance in the high-salinity group compared to the low-salinity group. It is important to note that 16S rRNA sequencing has limitations in genus-level classification, making it challenging to accurately distinguish between *Yoonia* and *Loktanella*. *Paracoccus* species are known to be halophilic or moderately halophilic bacteria (Kelly et al., 2006). For instance, *Paracoccus saliphilus*, isolated from a salt lake in Xinjiang, China, can grow in salinities ranging from 10‰ to 150‰, with optimal growth at 80‰ (Wang et al., 2009). Similarly, *Yoonia* and *Loktanella* have been isolated from salt lakes with salinity levels of 2.7‰ (Feng and Xing, 2023). All three genera belong to the family Rhodobacteraceae, a group primarily found in marine environments and often associated with phytoplankton, macroalgae, and marine animals (Wirth and Whitman, 2018). In the gut of Pacific white shrimp, Rhodobacteraceae constitutes a core bacterial group and may function as a probiotic (Dong et al., 2023). *Enterobacter*, another dominant genus in the high-salinity group, is commonly regarded as a halotolerant plant growth-promoting rhizobacterium. Some species can thrive at salinities as high as 60‰ (Pérez-Rodriguez et al., 2022) and can tolerate salinities up to 151‰ (Kapoor et al., 2017). In contrast, the dominant genera in the low-salinity group are less tolerant of high salinity; they cannot survive in environments with salinities exceeding 10‰, or their growth rates decline significantly (Tonk et al., 2007; Yoon et al., 2007; Zhang et al., 2020; Li et al., 2023). For example, *Tabrizicola* can tolerate high salinity but grows best in freshwater environments (Han et al., 2020), while *Microcystis* can survive in salinities up to 4‰ but is inhibited under conditions above 2‰ (Zhang et al., 2013). Therefore, the dominant bacteria in the high-salinity ZA community are highly salt-tolerant and thrive in such environments, while those in the low-salinity group are adapted to low-salinity or freshwater conditions. Consequently, salinity is the main factor driving changes in ZA community structure.

The relative abundance of Proteobacteria in the high-salinity group of ZA was significantly higher than in the low-salinity group. This finding aligns with previous research indicating that Proteobacteria thrive in various high-salinity ecological environments (Zhang et al., 2006; Xie et al., 2017). However, no significant difference in Proteobacteria abundance was observed between groups in FL and PA, possibly due to sample size limitations. At the genus level, *Paracoccus* exhibited the highest relative abundance in ZA, while *Cyanobium* PCC 6037 dominated in FL and PA. This phenomenon may be attributed to salinity reducing the diversity of ZA and enhancing competition among salt-tolerant *Paracoccus*. Additionally, previous studies have shown that the gut of zooplankton is typically anaerobic; for instance, the gut of copepods remains anaerobic even when external oxygen levels are high (Glud et al., 2015). *Paracoccus* can grow anaerobically in the presence of NO_3_-N or NO_2_-N (Nokhal and Schlegel, 1983), which may be another reason for its dominance in ZA compared to FL and PA. Moreover, DS001, a genus within Microbacteriaceae, had a lower relative abundance than *Cyanobium* PCC 6307 in FL and PA, and showed no significant advantage in ZA. This may be because members of Microbacteriaceae are primarily aerobic, with few exhibiting microaerophile or facultative anaerobiosis (Evtushenko, 2015), rendering DS001 less capable of adapting to the anaerobic conditions of the intestine.

As salinity increases, the relative abundance of salt-tolerant bacteria rises, while the relative abundance of bacteria less suited to high-salinity environments declines. Consequently, the functions contributed by these bacteria, whose relative abundances differ between groups, also vary. In the low-salinity group, characterized by higher Chl-*a* levels, there is a greater relative abundance of *Cyanobium* PCC 6307, leading to significant enrichment of functions associated with this genus. Unlike salinity, Chl-*a* does not directly influence these functions; rather, it reflects the high relative abundance of *Cyanobium* PCC 6307. In the low-salinity group, the functions that ZA, FL, and PA excel in are all related to phototrophy, which is attributed to *Cyanobium* PCC 6307. Conversely, in the high-salinity group, ZA and PA demonstrate significant advantages in nitrogen reduction, dark hydrogen oxidation, and methanol oxidation. These functions are primarily associated with *Paracoccus*, which aligns with the findings that the abundance of *Paracoccus* in ZA and PA varied significantly between different salinity groups, while no significant differences were observed in FL. Thus, because different bacteria exhibit varying tolerances to salinity, salinity influences the ZA community structure, thereby indirectly affecting the biochemical functions dominated by these bacteria. In addition, the majority of species in the genus *Paracoccus* are capable of using nitrate and its reduction products as alternative electron acceptors during anaerobic respiration (Katayama et al., 1995). The relative abundance of *Paracoccus* in high-salinity ZA was significantly higher than in low-salinity lakes. Therefore, in high-salinity lakes, the zooplankton gut may provide a suitable environment for *Paracoccus* to carry out denitrification, highlighting the ecological significance of ZA in saline lakes. This also indicates that the impact of salinity on ZA may further influence the ecological functions of zooplankton.

Our results indicate that salinity is the primary factor influencing the gut bacterial community and inferred functional composition of zooplankton in the Inner Mongolian Lake ecosystem. Due to varying bacterial tolerances to salinity, changes in salinity alter the community structure of ZA, reduce bacterial diversity and abundance, and indirectly affect the biochemical functions attributed to these bacteria, which aligns with our hypothesis. Under salinity stress, the community structure of ZA has changed, which may impact the ecological role of zooplankton in saline lakes. However, this impact requires further experimental validation. This study is the first to explore the response of ZA to environmental salinity, including changes in community structure and inferred functional composition. It also highlights the potential ecological significance of ZA, such as denitrification in high-salinity lakes. However, it is limited by the fact that zooplankton cannot adapt to excessively high salinities. Further research across a wider range of salinities and zooplankton species is necessary to verify the effects of salinity on the structure and functional expression of ZA communities.

## Data Availability

The raw sequencing data generated in this study have been deposited in the NCBI Sequence Read Archive (SRA) under the BioProject accession number PRJNA1188379.

## Footnotes

^1^ http://www.ehbio.com/ImageGP/index.php/Home/Index/FAPROTAX.html

^2^ https://github.com/huyanggroup/ggcor

^3^ https://www.r-project.org
