# Supplementary material for: Salinity-influenced changes in the community and functional composition of zooplankton-associated bacteria in the lakes of Inner Mongolia
Source: Front Microbiol. 2025 Jun 17;16:1529512. doi: 10.3389/fmicb.2025.1529512 (PMC12209351; doi:10.3389/fmicb.2025.1529512)
Supplement: Supplementary file 1 [file Supplementary_file_1.pdf]

Supplementary Material

1 Supplementary Figures

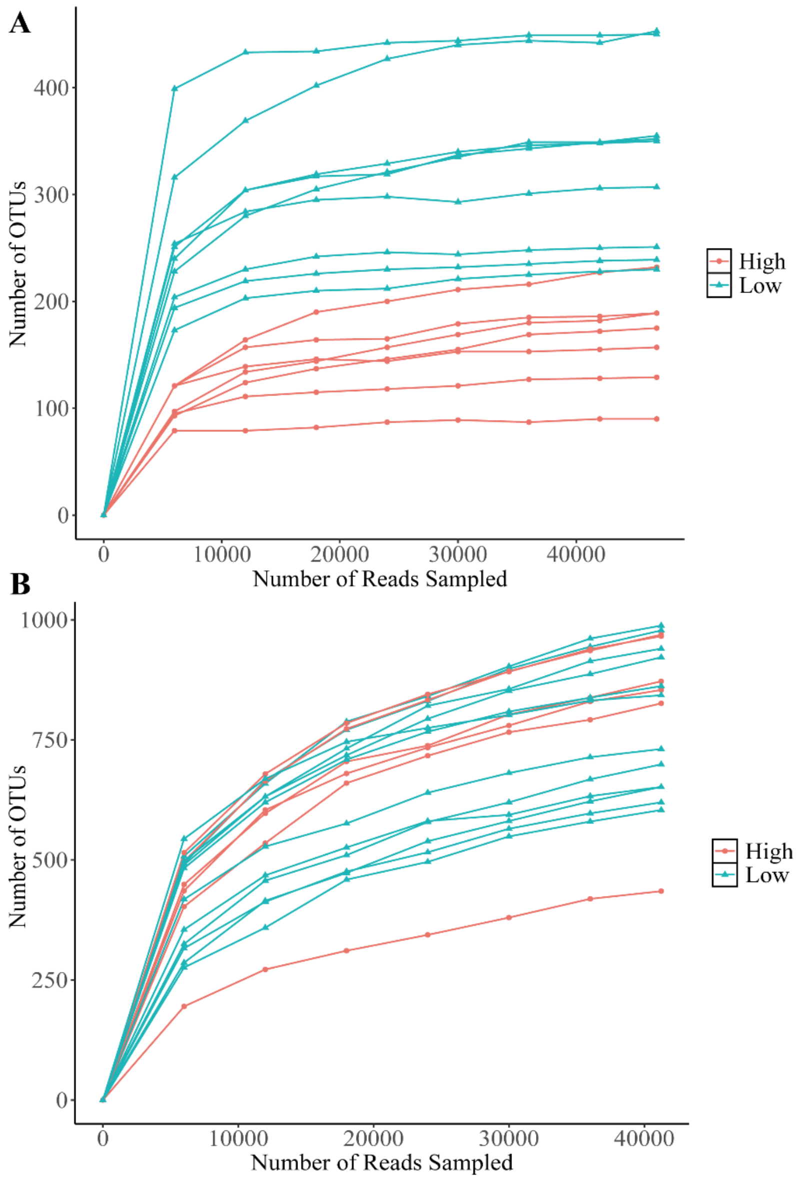

Supplementary Figure 1. Rarefaction curves were generated for the (A) 16 samples of zooplankton-associated bacteria and (B) 18 samples of free-living and particle-associated bacteria. The x-axis represents the amount of randomly selected sequencing data, while the y-axis indicates the number of OTUs.

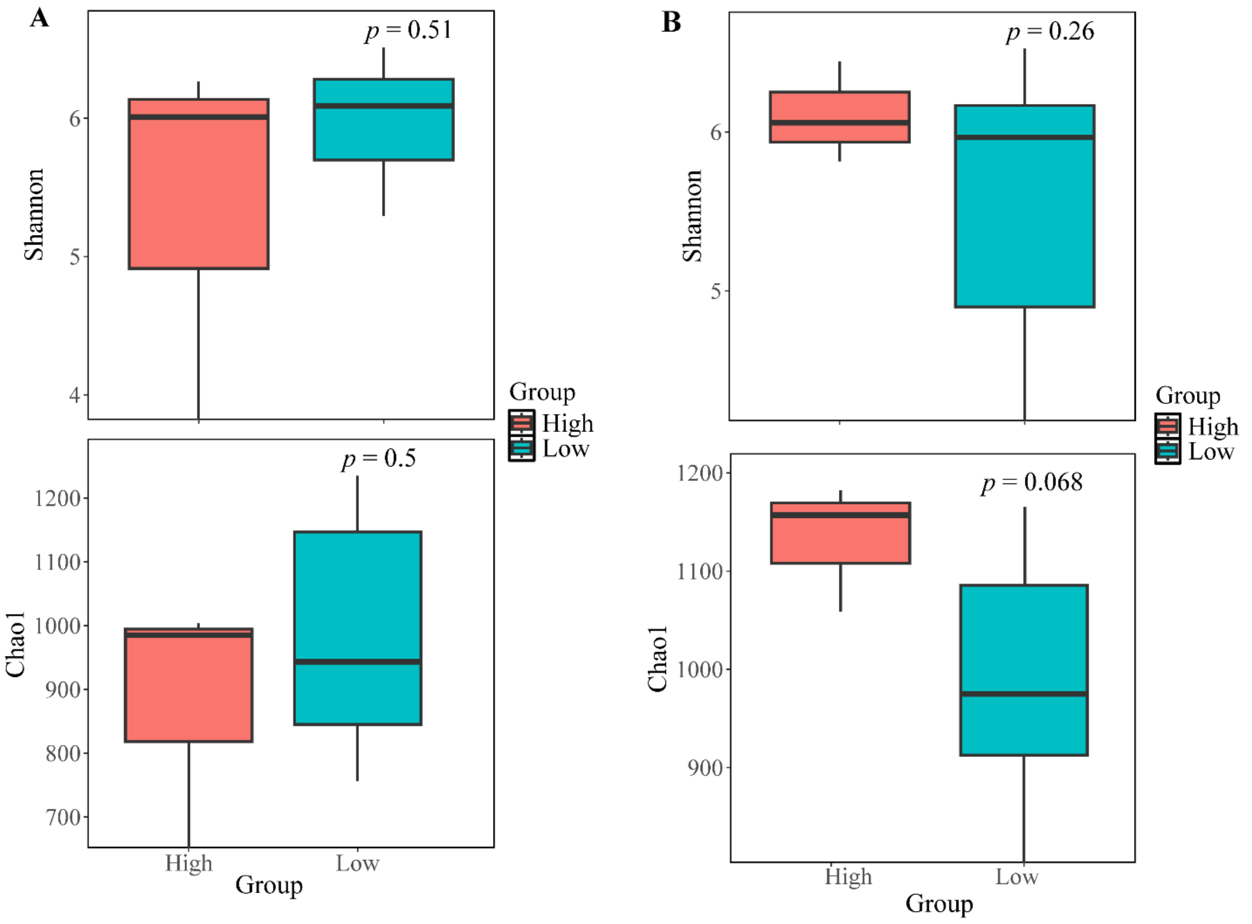

Supplementary Figure 2. Boxplot comparison of α-diversity (Shannon index and Chao1 index) among the four groups of (A) free-living bacteria and (B) particle-attached bacteria. The t-test was conducted to assess the significance of differences between groups.

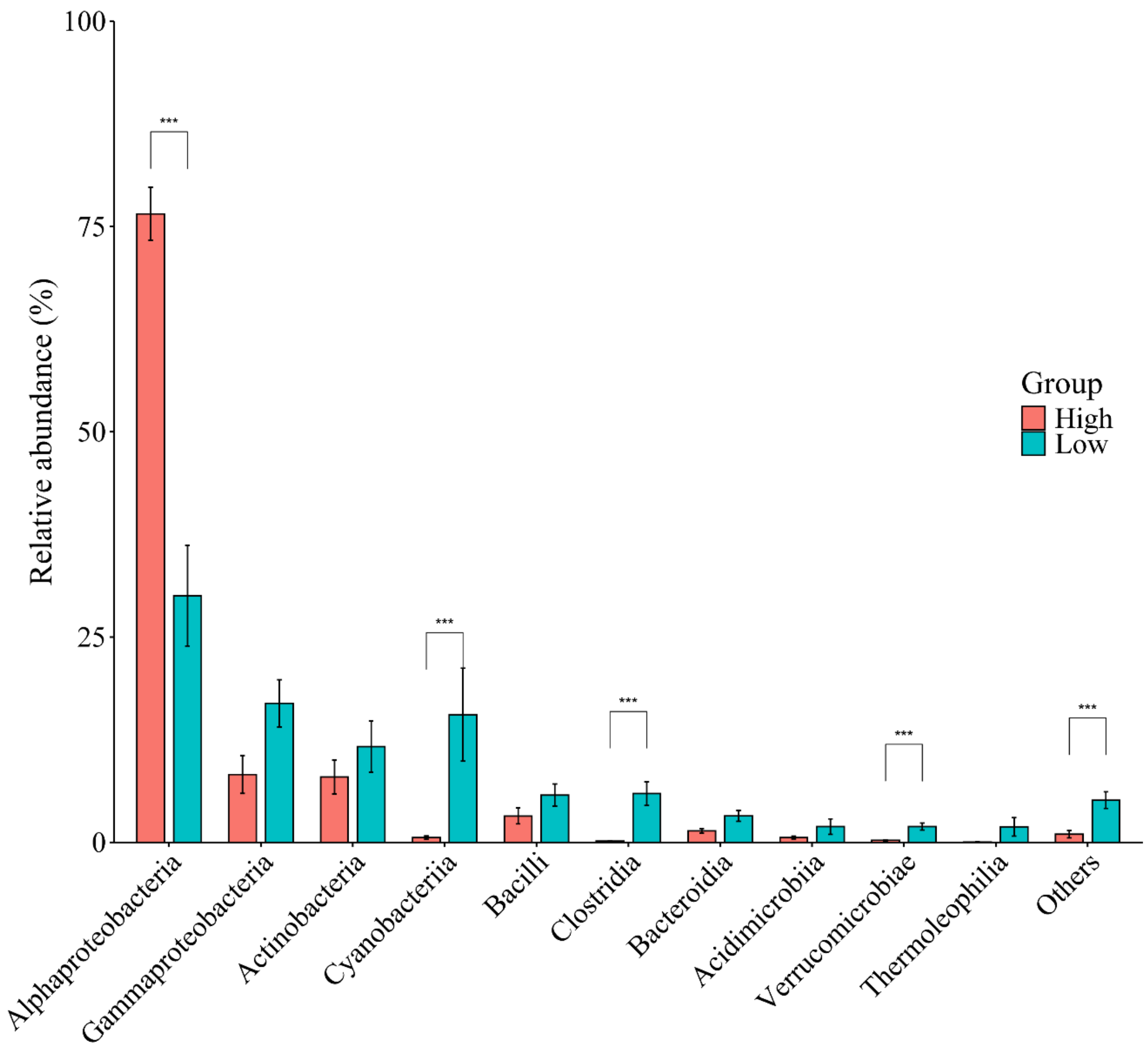

Supplementary Figure 3. Barplot showing the relative abundance of community composition in zooplankton-associated bacteria (ZA) at the class level (Top 10). Analysis was based on Wilcoxon rank-sum tests. Significance levels: *** *p*-value < 0.001.

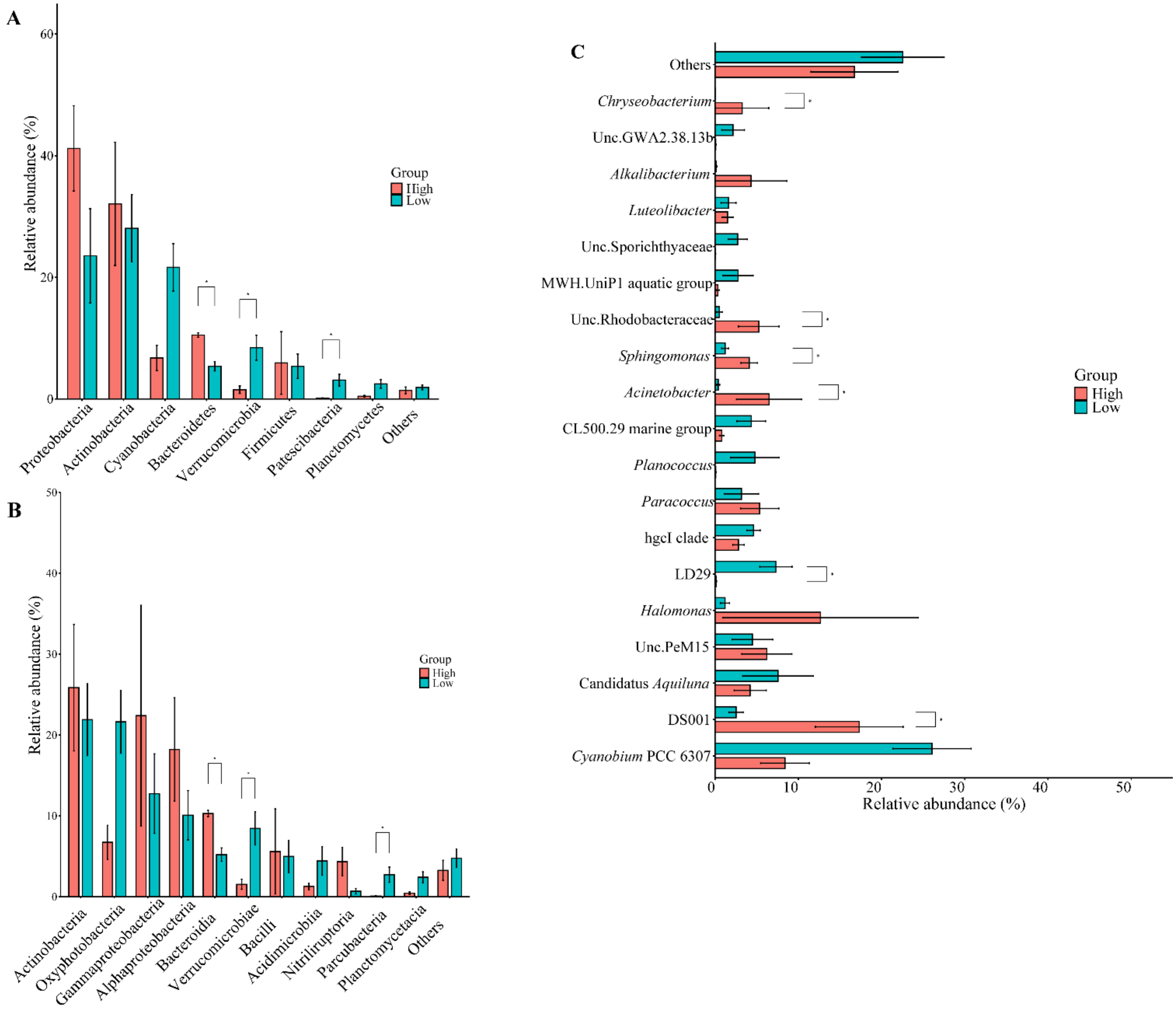

Supplementary Figure 4. Barplot showing the relative abundance of community composition in free-living bacteria (FL) at (A) the phylum level (Top 6), (B) the class level (Top 11) and (C) the genus level (Top 19). Analysis was based on Wilcoxon rank-sum tests. Significance levels: * *p*-value < 0.05.

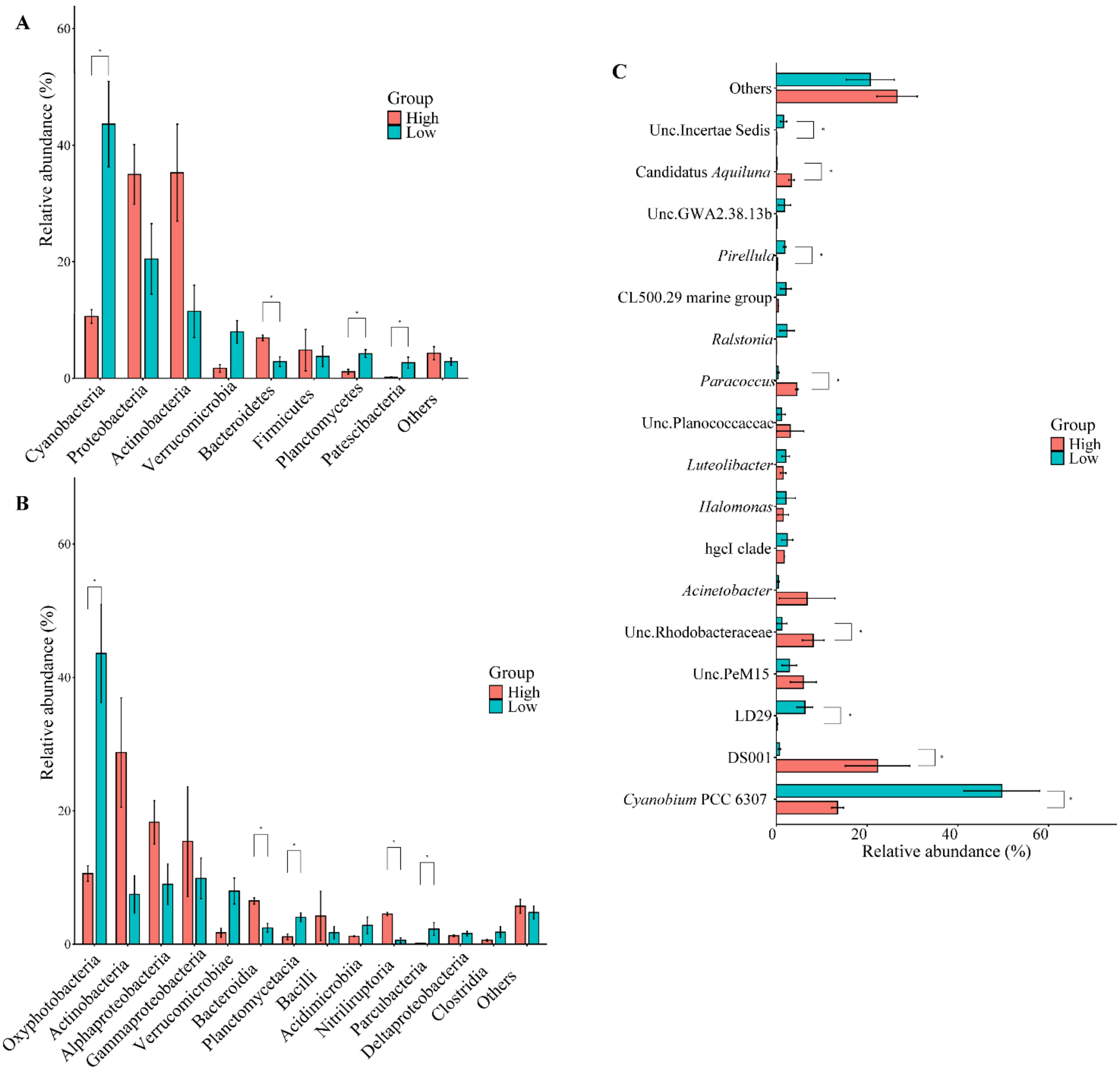

Supplementary Figure 5. Barplot showing the relative abundance of community composition in particle-associated bacteria (PA) at (A) the phylum level (Top 8), (B) the class level (Top 13) and (C) the genus level (Top 17). Analysis was based on Wilcoxon rank-sum tests. Significance levels: * *p*-value < 0.05.

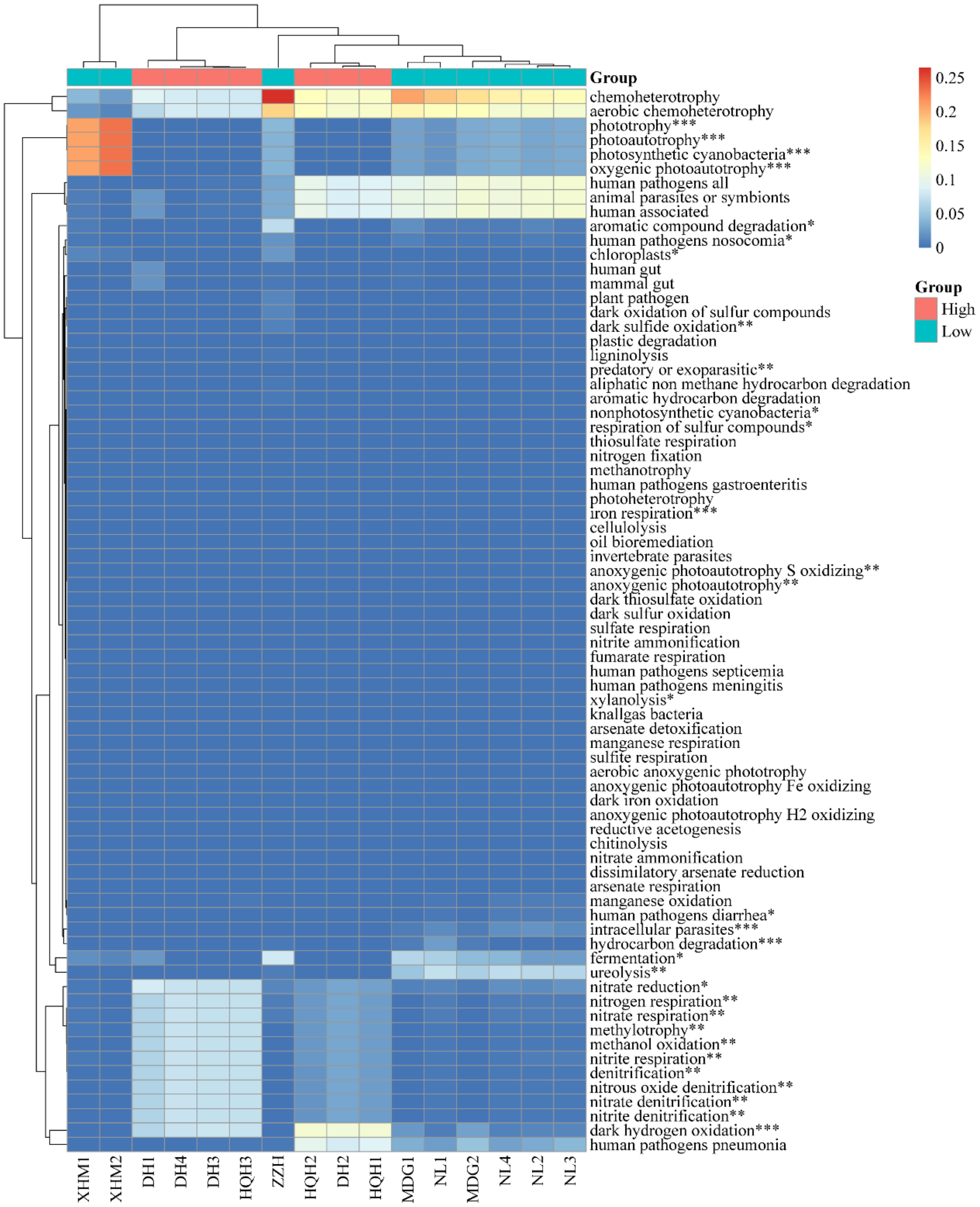

Supplementary Figure 6. Heatmap analysis of the relative abundance of predicted functions of zooplankton-associated bacteria across samples from high- and low-salinity groups. The Kruskal-Wall test was performed to assess significant differences in each function among different groups: *$p < 0.05$, **$p < 0.01$, ***$p < 0.001$.

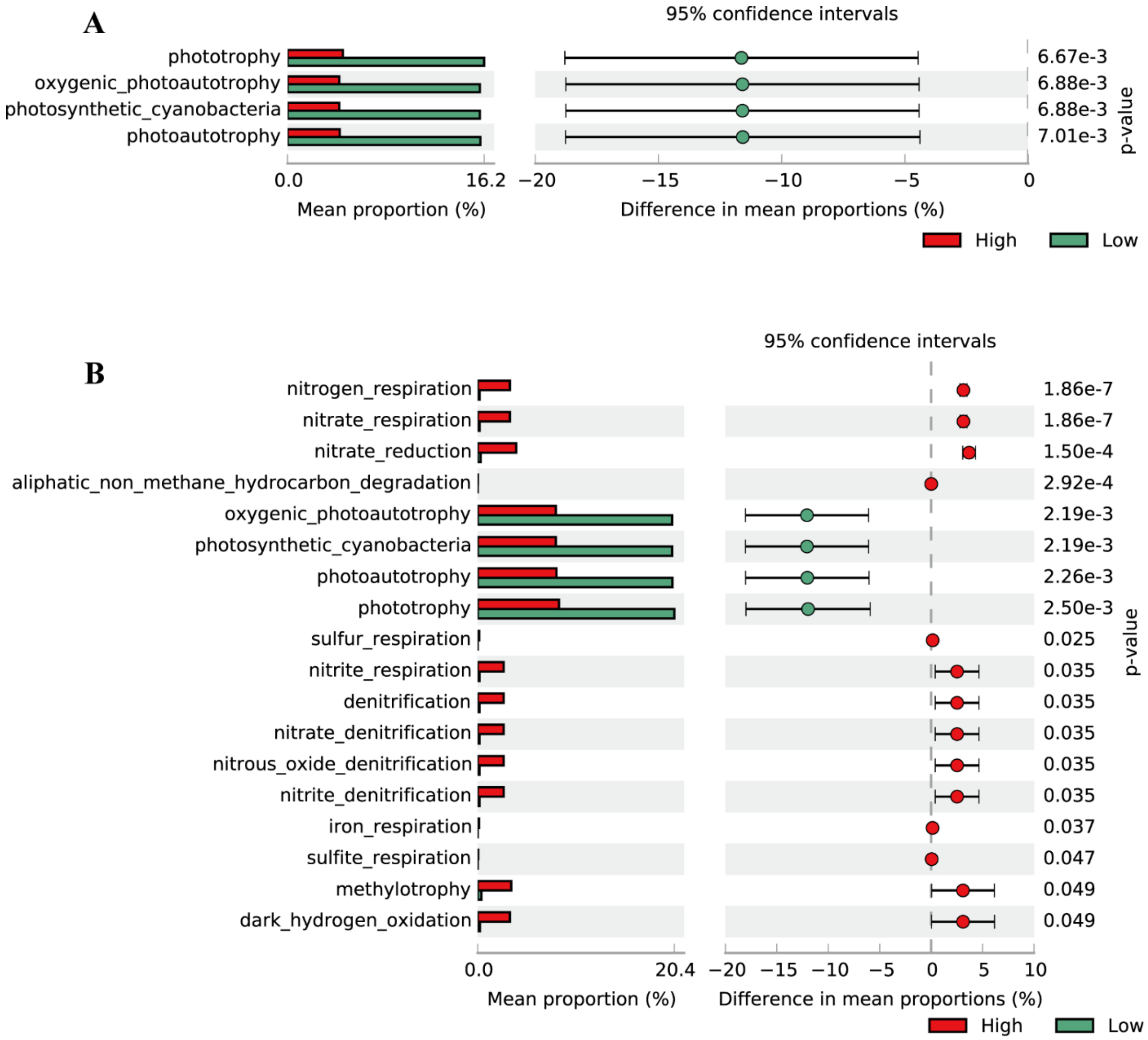

Supplementary Figure 7. Difference in the functional distribution of (A) free-living bacteria and (B) particle-attached bacteria based on FAPROTAX function predictions among high- and low-salinity groups. The analysis was conducted using Welch's t-test.

2 Supplementary Table

Supplementary Table 1. Characteristics of the six lakes in this study.

Lake (abbreviation)	Altitude	Salinity group	Mean salinity	Mean Chl-a	Mean NH_4-N	Mean NO_3-N	Mean NO_2-N	Mean PO_4-P	Mean TN	Mean TP
	m		ppt	μg/L	mg/L	mg/L	mg/L	mg/L	mg/L	mg/L
Nalin lake (NL)	986	Low	2.00	6.61	0.26	0.14	0.0016	0.007	0.86	0.08
Zhangzonghaizi (ZZH)	1407	Low	3.00	9.43	0.22	0.15	0.005	0.006	0.60	0.03
Maodonggou reservoir (MDG)	1427	Low	2.00	78.54	0.15	0.11	0.005	0.005	2.74	0.05
Xiaohamarigetainor (XHM)	1200	Low	2.38	279.13	0.37	0.2	0.11	0.433	6.66	1.06
Daihai (DH)	1195	High	17.00	4.20	0.14	0.23	0.001	0.034	6.86	0.33
Huangqihai (HQH)	1253	High	17.00	45.90	0.48	0.26	0.154	0.927	9.82	1.80
